# Nonconforming gender expression and insufficient sleep among adolescents during COVID-19 school closure and after school reopening

**DOI:** 10.1186/s12889-022-14463-4

**Published:** 2022-11-07

**Authors:** Qiguo Lian, Chaohua Lou, Xiangyang Zhong, Jiashuai Zhang, Xiaowen Tu, Yuhang Fang, Chunyan Yu, Xiayun Zuo

**Affiliations:** 1 NHC Key Lab. of Reproduction Regulation (Shanghai Institute for Biomedical and Pharmaceutical Technologies), Fudan University, Shanghai, 200237 China; 2 Shanghai Jing’an Education College, Shanghai, 200070 China; 3 School of Public Health, Fudan University, Shanghai, 200032 China

**Keywords:** Gender nonconformity, Insufficient sleep, COVID-19

## Abstract

**Background:**

Gender nonconformity (GNC) (i.e., gender expression that differs from gender role expectations for feminine or masculine appearance and behavior) is an under-researched area of adolescent sleep health. The COVID-19 lockdown offers an opportunity to understand how the effect of GNC on adolescent health outcomes changes between school closure and reopening.

**Methods:**

We conducted a cross-sectional study in Shanghai, China, in 2020. The sample size for analysis was 3,265. The age-specific insufficient sleep was estimated according to National Sleep Foundation's sleep duration recommendations. The self-perceived and self-rated GNC were measured by the two items “On the same scale that goes from 100% as a girl to 100% as a boy, where do you think others see you?” and “On a scale that goes from feeling 100% like a girl to feeling 100% like a boy, where do you see yourself?”, and birth sex. In addition, we calculated sex-stratified adjusted odds ratios (AORs) of insufficient sleep for students with high and moderate GNC compared to students with low GNC. Finally, we measured the AORs with self-perceived and self-rated GNC during COVID-19 school closure and reopening.

**Results:**

Among 3,265 students in grade 6–12 in the analytic sample, 1,567(48.0%) were assigned female at birth (AFAB), 3,188 (97.6%) Han, and 1,921(58.8%) in grade 6–9. Among AFAB students, high self-perceived GNC was significantly associated with insufficient sleep (AOR,1.65; 95%CI,1.30–2.09) during school closure. Insufficient sleep was associated with high self-rated GNC (AOR,1.73; 95%CI,1.23–2.44) and moderate self-rated GNC (AOR,1.69; 95%CI,1.29–2.22) during school closure. After school reopening, neither self-perceived nor self-rated GNC was associated with insufficient sleep among AFAB students. Among assigned male at birth (AMAB) students, none of the two kinds of GNC was associated with insufficient sleep in the two periods during the COVID-19 pandemic.

**Conclusions:**

This study suggests GNC is only associated with insufficient sleep among AFAB students during school closure. Furthermore, the association is nonsignificant among AMAB students. These findings indicate that GNC-related stigma within the family could be a risk factor for insufficient sleep among AFAB adolescents.

## Introduction

Adolescent sleep health has been recognized as a global priority [1]. Insufficient sleep (short sleep duration) poses a serious threat to this age group's academic success, physical and mental health, and road safety [2, 3]. Insufficient sleep is predominant among adolescents worldwide in modern society. In the United States, the prevalence of adolescents with no more than 7 h of sleep has increased across ages from 1991 to 2012 [4]. Another trend analysis based on the Youth Risk Behavior Survey (YRBS) indicated a significant linear increase (68.9%-74.6%) in the prevalence of sleep duration below 8 h during 2007–2017 in U.S. high school students in grades 9–12 [5]. Similarly, in Japan, the Lifestyle Survey of Adolescents study found that the national prevalence of sleep duration below 7 h increased from 71.9% in 2004 to 78.1% in 2017 in students in grades 7–12 [6]. In China, no national trend data on insufficient sleep among adolescents were reported; the prevalence was 77.2% in students aged 9–18 in the 2014 Chinese National Survey on Students' Constitution and Health (CNSSCH) [7].

Minority stress is an apparent risk factor for ill-health, especially among adolescents, and GNC is a typical example. GNC describes the degree of discrepancy between an individual’s gender expression (e.g., style of hair or dress, voice, or movement) and the gender role expectations (e.g., the cultural roles expected of adolescent females and males based on their sex) [8, 9]. Gender expression can be perceived from multiple perspectives, such as self-perceived gender expression (how a person perceives their gender to be viewed by others [10]) and self-rated gender expression (how a person rates subjectively their femininity and masculinity [10]). Evidence shows that gender nonconforming youths are at increased risk of peer violence and child maltreatment [11–14]. Also, gender nonconformity (GNC) and its associated health outcomes may vary by sex [8, 15]. The 2015 YRBS indicated that High GNC was more prevalent among male students than female students [8]. In addition, the 2013 YRBS showed significant differences by GNC in disordered weight-control behaviors among male students but not female students [15]. The minority stress model [16] posits that exposure to GNC-induced stigma or stress may elevate the risk of many health outcomes as a coping response [8, 12, 13, 17, 18]. This stress may contribute significantly to insufficient sleep among adolescents [4], and emerging research suggests that GNC may be a source of stress [19]. Therefore, we expect that adolescents' perceived GNC-induced stigma might impact their sleep duration. However, no studies to date have examined GNC as a risk factor for adolescent insufficient sleep and how this association may vary by sex.

The coronavirus disease 2019 (COVID-19), first reported in late December 2019, has become a global pandemic [20]. In response to the COVID-19 pandemic, lots of cities issued lockdown and stay-at-home orders. Many studies reported that adolescents sleep longer during COVID-19 school closure [21–23]. The COVID-19 lockdown creates an uncommon scenario for adolescents living with their family members all days, and it provides an opportunity to examine how the effect of GNC on insufficient sleep changes during and after the COVID-19 lockdown. To take advantage of the natural experiment (a shift in exposure [gender expression related stress] that was caused by forces outside the researchers’ control [24]), we conducted a cross-sectional study to examine the association between GNC and insufficient sleep across sex by collecting the information of GNC and sleep duration during school closure and after school reopening.

## Methods

### Study design and participants

The COVID-19 pandemic caused a widespread lockdown in many cities across China in 2020, including Shanghai. In response to the COVID-19 pandemic, Shanghai authorities closed the schools and offered e-courses over the internet to all the students since March 11. The schools reopened between April 27 and June 2, 2020, for all students in grades 6–12.

We used a cross-sectional study design to assess sleep duration during and after the lockdown in 2020 in Shanghai. In coordination with the education authorities, we undertook a one-time, anonymous online survey in two junior high schools and four combined junior and senior high schools between June 28 and July 13, 2020, in Shanghai, China. Of the 3,564 students aged 10 to 20 years, 299 declined to participate, leading to a response rate of 91.6%.

### Ethical approval and consent to participate

With the help of the school teachers, we sent out digital informed consent to the parents or guardians of all students a week before the survey during the ongoing COVID-19 pandemic. They were requested to send back an electronically signed form only if they did not allow their child to participate in the survey. We obtained active consent from the students by asking if they agreed to participate in the first question voluntarily. They would skip to the end of the survey if they answered “No” to the question. The ethical committee of the Shanghai Institute of Planned Parenthood Research reviewed and approved this study (PJ2020-26).

## Measures

### Outcome variable

We measured sleep duration by collecting the bedtime and wake-up time on normal weekdays during school closure and after school reopening using the four questions [21]: “When do you usually go to bed on weekdays during school closure?”, “When do you usually wake up on weekdays during school closure?”, “When do you usually go to bed on weekdays after school reopening?” and “When do you usually wake up on weekdays after school reopening?” The options for bedtime ranged from “No later than 18:00” to “1:00 or later” in half-hour intervals, and for wake-up time ranged from “No later than 5:00” to “10:00 or later” with a half-hour interval.

In calculations of sleep duration, we used the midpoint time for each half-hour interval (e.g., 21:15 for “21:01–21:30”) and the minimum or maximum time at the ends of the options (e.g., 18:00 for “No later than 18:00” and 1:00 for “1:00 or later”) [21, 25]. We then labeled the students as insufficient sleep if they slept less than the recommended sleep duration for their age according to National Sleep Foundation (at least 9 h for school-age children aged 5–13 years, 8 h for teenagers aged 14–17 years, and 7 h for young adults aged 18–25 years) [26].

### Exposure variables

We measured both self-perceived and self-rated gender expression, given that discrepancies may exist between them. Using one item adapted from a validated measure [27], we assessed self-perceived gender expression with the following statement and question: “On the same scale that goes from 100% as a girl to 100% as a boy, where do you think others see you?” Response options were “They see me 100% like a girl,” “They see me more like a girl,” “They see me in the middle,” “They see me more like a boy,” and “They see me 100% like a boy.”

Similarly, we measured self-rated gender expression with the following statement and question: “On a scale that goes from feeling 100% like a girl to feeling 100% like a boy, where do you see yourself?” Response options were “I feel 100% like a girl,” “I feel more like a girl,” “I feel in the middle,” “I feel more like a boy,” and “I feel 100% like a boy.”

We created two 5-point GNC scores based on a student’s response to self-perceived and self-rated gender expression and to the question “What is your sex?” (“Female” (assigned female at birth, AFAB) or “Male” (assigned male at birth, AMAB)). Students were categorized from most gender conforming (1, indicating 100% feminine females and 100% masculine males) to most gender nonconforming (5, indicating 100% masculine females and 100% feminine males). Based on the GNC scores, we then generated two 3-level GNC variables (self-perceived and self-rated GNC) respectively: (1) low GNC (GNC score:1-2), (2) moderate GNC (GNC score: 3), and (3) high GNC (GNC score: 4-5).

### Covariates

According to prior research on GNC and health outcomes [8, 28], we included individual-level characteristics as control variables, including sex (AFAB or AMAB), ethnicity (Han or non-Han), and grade (6–12).

### Statistical analysis

We used Stata/SE 15.1 (StataCorp LLC, College Station, TX, USA) for all the data analyses. We calculated the prevalence estimates of self-perceived and self-rated GNC and insufficient sleep with 95% confidence interval (CI). Differences in self-perceived and self-rated GNC distribution and insufficient sleep by demographic characteristics were examined using chi-square statistics. Given that self-perceived and self-rated GNC and insufficient sleep tend to vary by sex, we estimated the sex-stratified adjusted odds ratios (AOR) and 95% CI for all associations of self-perceived and self-rated GNC with insufficient sleep after adjusting for ethnicity and grade using logistic regression models with robust standard errors for clustering by levels of participating schools. The level of statistical significance was set at a 2-sided *P* < 0.05.

### Role of the funding source

The funders had no role in the study design, data collection, analysis, publication decision, or manuscript preparation.

## Results

In total, this study analyzed 3,265 students with a mean (standard deviation) age of 14.6 (1.99) years in grades 6–12. There were 1,567(48.0%) AFAB students, 3,188 (97.6%) Han students, and 1,921(58.8%) students in grade 6–9 in the analytic sample.

Self-perceived GNC varied by sex and grade group (Table 1). High self-perceived GNC was more prevalent among AFAB students (14.5%) than AMAB students (4.0%) and was more prevalent among students in grades 10–12 (10.6%) than those in grades 6–9 (8.0%). Self-rated GNC showed a similar distribution pattern (Table 2). The report of high self-rated GNC was statistically significantly higher among female students (8.6%) than male students (2.0%) and was statistically significantly higher among students in grades 10–12 (6.2%) than those in grades 6–9 (4.4%).

**Table 1:** Self-perceived gender nonconformity among students by sex, ethnicity, and school type

Demographic characteristic	Self-perceived gender nonconformity, % (95% CI)			χ^2^ *P* Value
	**Low**	**Moderate**	**High**	
**Total population**	73.6(72.1–75.1)	17.4(16.1–18.7)	9.0(8.1–10.1)	N.A
**Sex**				
AFAB	61.3(58.9–63.7)	24.2(22.1–26.4)	14.5(12.8–16.3)	< 0.001
AMAB	84.9(83.1–86.5)	11.1(9.7–12.7)	4.0(3.2–5.0)	
**Ethnicity**				
Han	73.6(72.1–75.1)	17.3(16.1–18.7)	9.0(8.1–10.1)	0.981
Non-Han minorities	72.7(61.8–81.5)	18.2(11.1–28.4)	9.1(4.4–17.9)	
**Grade group**				
Junior high school (grade 6–9)	77.0(75.1–78.8)	15.0(13.5–16.7)	8.0(6.8–9.3)	< 0.001
Senior high school (grade 10–12)	68.8(66.2–71.2)	20.7(18.6–22.9)	10.6(9.0–12.3)	

*Abbreviation*: *CI* confidence interval, *N.A*. not applicable, *AFAB* assigned female at birth, *AMAB* assigned male at birth

**Table 2:** Self-rated gender nonconformity among students by sex, ethnicity, and school type

Demographic characteristic	Self-rated gender nonconformity, % (95% CI)			χ^2^ *P* Value
	**Low**	**Moderate**	**High**	
**Total population**	75.9(74.4–77.4)	18.9(17.6–20.3)	5.1(4.4–6.0)	N.A
**Sex**				
AFAB	61.3(58.8–63.6)	30.2(28.0–32.5)	8.6(7.3–10.0)	< 0.001
AMAB	89.5(87.9–90.8)	8.5(7.3–10.0)	2.0(1.4–2.8)	
**Ethnicity**				
Han	76.1(74.6–77.5)	18.9(17.5–20.2)	5.1(4.4–5.9)	0.399
Non-Han minorities	70.1(59.0–79.3)	22.1(14.2–32.7)	7.8(3.5–16.3)	
**Grade group**				
Junior high school (grade 6–9)	80.1(78.2–81.8)	15.5(14.0–17.2)	4.4(3.6–5.4)	< 0.001
Senior high school (grade 10–12)	70.0(67.5–72.4)	23.8(21.6–26.2)	6.2(5.0–7.6)	

*Abbreviation*: *CI* confidence interval, *N.A.* not applicable, *AFAB* assigned female at birth, *AMAB* assigned male at birth

Table 3 shows the prevalence of insufficient sleep during the school closure and after school reopening among students by demographic subgroups. We observed that insufficient sleep during the school closure did not vary by demographic characteristics, including sex, ethnicity, and grade. However, insufficient sleep after school reopening did vary by sex and grade group. For example, self-reported insufficient sleep during the school closure was statistically significantly higher among AFAB students than AMAB students (70.3% versus 58.2%) and was statistically significantly higher among students in grades 10–12 than those in grades 6–9 (67.4% versus 61.6%).

**Table 3:** Insufficient sleep among students by sex, ethnicity, and school type during school closure and after school reopening

Demographic characteristic	Insufficient sleep, % (95% CI)		χ^2^ *P* Value
	Yes	No	
**School closure**			
**Total population**	21.1(19.7–22.5)	78.9(77.5–80.3)	N.A
**Sex**			
AFAB	22.3(20.3–24.5)	77.7(75.5–79.7)	0.097
AMAB	20.0(18.1–21.9)	80.0(78.1–81.9)	
**Ethnicity**			
Han	21.2(19.8–22.6)	78.8(77.4–80.2)	0.525
Non-Han minorities	18.2(11.1–28.4)	81.8(71.6–88.9)	
**Grade group**			
Junior high school (grade 6–9)	20.5(18.8–22.4)	79.5(77.6–81.2)	0.321
Senior high school (grade 10–12)	21.9(19.8–24.2)	78.1(75.8–80.2)	
**School reopening**			
**Total population**	64.0(62.3–65.6)	36.0(34.4–37.7)	N.A
**Sex**			
AFAB	70.3(67.9–72.5)	29.7(27.5–32.1)	< 0.001
AMAB	58.2(55.8–60.5)	41.8(39.5–44.2)	
**Ethnicity**			
Han	64.1(62.4–65.7)	35.9(34.3–37.6)	0.586
Non-Han minorities	61.0(49.8–71.2)	39.0(28.8–50.2)	
**Grade group**			
Junior high school (grade 6–9)	61.6(59.4–63.7)	38.4(36.3–40.6)	0.001
Senior high school (grade 10–12)	67.4(64.9–69.9)	32.6(30.1–35.1)	

*Abbreviation*: *CI* confidence interval, *N.A.* not applicable, *AFAB* assigned female at birth, *AMAB* assigned male at birth

Table 4 shows adjusted associations of insufficient sleep during the school closure and after school reopening with self-perceived GNC, and Table 5 shows the adjusted associations with self-rated GNC. For AFAB students, insufficient sleep during the school closure was associated with high self-perceived GNC compared with those with low self-perceived GNC (AOR,1.65; 95% CI, 1.30–2.09; *P* < 0.001). During the school closure, insufficient sleep was associated with higher self-rated GNC than low self-rated GNC among AFAB students (AOR,1.73; 95% CI, 1.23–2.44; *P* = 0.002). Also, insufficient sleep was associated with moderate self-rated GNC compared with those with low self-rated GNC among AFAB students (AOR, 1.69; 95% CI, 1.29–2.22; *P* < 0.001). After school reopened, none of the GNC was associated with insufficient sleep among AFAB students. For AMAB students, we did not observe any association of insufficient sleep during the school closure and after school reopening with self-perceived and self-rated GNC.

**Table 4:** Sex-specific associations of insufficient sleep with self-perceived gender nonconformity among students during school reopening and after school reopening

Insufficient sleep	Self-perceived gender nonconformity						
	**Low**	**Moderate**			**High**		
	**%**	**%**	**AOR (95% CI)**	**P value**	**%**	**AOR (95% CI)**	***P* value**
**AFAB Students**							
School closure	20.3	23.5	1.21(0.90–1.63)	0.203	29.1	1.65(1.30–2.09)	< 0.001
School reopening	70.8	69.7	0.91(0.64–1.31)	0.628	69.2	0.90(0.56–1.47)	0.684
**AMAB Students**							
School closure	20.0	18.6	0.89(0.55–1.43)	0.626	23.5	1.20(0.73–1.97)	0.478
School reopening	58.3	56.4	0.88(0.71–1.08)	0.223	60.3	1.06(0.65–1.75)	0.805

*Abbreviation*: *AOR* adjusted odds ratio (adjusted for ethnicity, and grade, with low gender nonconformity being the referent group), *CI* confidence interval, *AFAB* assigned female at birth, *AMAB* assigned male at birth. We did not observe any difference of insufficient sleep between moderate and high gender nonconformity students

**Table 5:** Sex-specific associations of insufficient sleep with self-rated gender nonconformity among students during school reopening and after school reopening

Insufficient sleep	Self-rated gender nonconformity						
	**Low**	**Moderate**			**High**		
	**%**	**%**	**AOR (95% CI)**	***P* value**	**%**	**AOR (95% CI)**	***P* value**
**AFAB Students**							
School closure	19.0	27.5	1.69(1.29–2.22)	< 0.001	28.4	1.73(1.23–2.44)	0.002
School reopening	70.0	70.2	0.98(0.65–1.47)	0.922	72.4	1.09(0.74–1.61)	0.662
**AMAB Students**							
School closure	19.7	22.8	1.17(0.77–1.78)	0.465	17.6	0.88(0.31–2.52)	0.817
School reopening	58.0	59.3	0.99(0.77–1.28)	0.962	61.8	1.22(0.71–2.12)	0.468

*Abbreviation*: *AOR* adjusted odds ratio (adjusted for ethnicity, and grade, with low gender nonconformity being the referent group), *CI* confidence interval, *AFAB* assigned female at birth, *AMAB* assigned male at birth. We did not observe any difference of insufficient sleep between moderate and high gender nonconformity students

## Discussion

This study, to our knowledge, is the first to investigate associations between GNC measured in two dimensions and insufficient sleep among adolescents using two different scenarios of school status (school closure versus school reopening) during the ongoing COVID-19 pandemic. The study design provides a unique and great opportunity to examine how the change of GNC-related stress the adolescents experienced (staying at home all day with parents and family members during school closure versus normal social lives as a student after the school reopening) may affect their sleep health.

Within our study population, about 1 in 4 students reported either moderate (17.4%) or high (9.0%) levels of self-perceived GNC and reported either moderate (18.9%) or high (5.1%) levels of self-rated GNC. The findings are higher than those of a study in the United States, which reported a prevalence of 11.9% and 8.4% for moderate and high levels of self-perceived GNC [8]. And the data on self-rated GNC among adolescents are scarce. In line with previous studies [8, 29], the prevalence of self-perceived and self-rated GNC varied by sex and grade group.

Insufficient sleep among adolescents has become a pervasive and prominent health problem worldwide. The objectives for sleep health in Healthy People 2030 specifically include reducing adolescent sleep loss: “Increase the proportion of high school students who get enough sleep — SH‑04” [30] (defined as ≥ 8 h for students in grades 9 through 12). Similarly, Healthy China Action Plan 2019–2030 recommends at least 10 h for children in primary school, 9 h for those in junior high school, and 8 h for those in senior high school [31]. However, up to 74.6% of U.S. high school students got insufficient sleep on weekdays in 2017 YRBS [5], and the prevalence was 77.2%% among children and adolescents aged 9–18 years in the 2014 CNSSCH [7]. In our study, the prevalence of insufficient sleep was also very high (64.0%) after school opening. However, we found the prevalence decreased sharply decreased by two-thirds during the COVID-19 school closure [21]. In our previous research, we attributed this change partly to zero school commuting time, lower academic stress, and the closure of private educational institutions during the COVID-19 lockdown [21].

There were differences among AFAB students in the associations between GNC and insufficient sleep during school closure and after school opening. During school closure, only students with high self-perceived GNC were more likely to report insufficient sleep than those with low self-perceived GNC. In contrast, students with moderate and high self-rated GNC were more likely to experience insufficient sleep than those with low self-rated GNC. These findings may add to the evidence supporting the unique challenges AFAB students face in the middle of the GNC spectrum [8]. After school reopening, no associations exist between insufficient sleep and self-perceived and self-rated GNC, which indicates that, at least in Shanghai, AFAB students may feel higher GNC-related stress from their family members than from people in their neighborhoods and schools.

Among AMAB students, self-perceived and self-rated GNC were not associated with insufficient sleep during school closure and the lack of associations held up after school opening. The findings suggest that insufficient sleep among AMAB students is immune from self-perceived and self-rated GNC. However, the findings are inconsistent with a recent study which reveals that parents are usually more comfortable with gender nonconforming girls than boys and are more likely to try to modify their sons’ behaviors [32]. The main reason for the discrepancy is that the study measured GNC from parental perspectives, which is unrelated to children's reported GNC-induced pressure [33].

Sleep may be a health disparity in sexual and gender minority individuals (i.e., lesbian, gay, bisexual, transgender, queer, or other identities) [34], and the potential mechanism underlying the observed sleep disparity is social and minority stress [16]. And social stress has a similar role regarding self-perceived and self-rated GNC. However, GNC's effects on sleep may differ with their effects on adolescent mental distress, substance use, and bullying [8, 13, 18, 35]. Therefore, more research is needed to understand deep the links between gender norms, minority stressors, and insufficient sleep across the GNC spectrum. Also, given that transgender adolescents (individuals whose gender identity does not align with their birth sex) and cisgender peers (individuals whose gender identity aligns with their birth sex) with GNC often have similar adverse experiences [8], it is important to investigate whether associations of insufficient sleep with transgender identity are similar to the findings with gender nonconforming students in this study. In our previous study, we found both similarities and differences in bullying between transgender identity and GNC among adolescents [13]. Unfortunately, we did not collect the data on transgender identity, hence could not identify transgender students in this study.

## Implications

Creating safe spaces for children and adolescents to explore their gender identity and gender expression may help buffer GNC-induced stresses and contribute to positive health outcomes, including sleep. Parents should recognize that family support is crucial to the health and wellbeing of their children (girls in particular) with GNC. School personnel should be equipped with adequate knowledge and skills to manage the issue of GNC in their daily interactions with families with gender nonconforming children [36, 37]. Public health researchers could partner to design and implement interventions that are inclusive of gender diversity in schools [38], and more research needs to focus on GNC and its effects on insufficient sleep. Pediatricians could also benefit from exploring gender identity, and GNC concerns confidential as part of routine adolescent health care when identifying potential causes of sleep disorders [39, 40].

## Limitations

This study is subject to some limitations. First, our study sample only included adolescents who attend school, and gender minority adolescents may be disproportionately high among school dropouts and truants [41]. Second, students may reluctant to reveal their true gender expression (i.e., GNC) because of social stigma and taboo, which could underestimate the strength of associations between self-perceived and self-rated GNC and insufficient sleep studied herein. Third, we collected the self-reported bedtime and wake-up time during the school closure and after school reopening retrospectively, which might introduce recall bias. Fourth, we investigated the association of GNC with insufficient sleep using the cross-sectional design, which indicates that causality cannot be guaranteed. Furthermore, the cross-sectional design could not allow us to examine the changes in gender norms on the individual and social levels. Fifth, our findings from Shanghai adolescents may not generalize directly to adolescents in other cities without considering the potential differences in social and gender norms between cities.

## Conclusions

Public health efforts of developing support systems (within families in particular) for gender nonconforming students may help improve sleep health and reduce insufficient sleep among adolescents, especially AFAB adolescents.

## Data Availability

The datasets used and/or analyzed during the current study are available from the corresponding authors on reasonable request.

## Abbreviations

AFAB: Assigned female at birth
AMAB: Assigned male at birth
AOR: Adjusted odds ratios
CI: Confidence interval
CNSSCH: Chinese National Survey on Students Constitution and Health
COVID-19: Coronavirus disease 2019
GNC: Gender nonconformity
YRBS: Youth Risk Behavior Survey

## Declarations

### Competing of interests

None declared.
